# Tuning carrier density and phase transitions in oxide semiconductors using focused ion beams

**DOI:** 10.1515/nanoph-2022-0050

**Published:** 2022-06-13

**Authors:** Hongyan Mei, Alexander Koch, Chenghao Wan, Jura Rensberg, Zhen Zhang, Jad Salman, Martin Hafermann, Maximilian Schaal, Yuzhe Xiao, Raymond Wambold, Shriram Ramanathan, Carsten Ronning, Mikhail A. Kats

**Affiliations:** Department of Electrical and Computer Engineering, University of Wisconsin-Madison, Madison, Wisconsin 53706, USA; Institute of Solid State Physics, Friedrich Schiller University Jena, Jena, 07743, Germany,Carsten.Ronning@uni-jena.de; Department of Materials Science and Engineering, University of Wisconsin-Madison, Madison, Wisconsin 53706, USA; School of Materials Engineering, Purdue University, West Lafayette, IN 47907, USA; Department of Physics, University of Wisconsin-Madison, Madison, Wisconsin 53706, USA

**Keywords:** defect engineering, doping, focused ion beam, mask-free lithography, vanadium dioxide (VO_2_), zinc oxide (ZnO)

## Abstract

We demonstrate spatial modification of the optical properties of thin-film metal oxides, zinc oxide (ZnO) and vanadium dioxide (VO_2_) as representatives, using a commercial focused ion beam (FIB) system. Using a Ga^+^ FIB and thermal annealing, we demonstrated variable doping of a wide-bandgap semiconductor, ZnO, achieving carrier concentrations from 10^18^ cm^−3^ to 10^20^ cm^−3^. Using the same FIB without subsequent thermal annealing, we defect-engineered a correlated semiconductor, VO_2_, locally modifying its insulator-to-metal transition (IMT) temperature by up to ∼25 °C. Such area-selective modification of metal oxides by direct writing using a FIB provides a simple, mask-less route to the fabrication of optical structures, especially when multiple or continuous levels of doping or defect density are required.

## 1 Introduction

Focused ion beam (FIB) is a well-established technique for high-resolution area-selective milling, deposition, and imaging [1–5]. For example, FIB-assisted deposition and milling has been broadly used for applications such as TEM specimen preparation [6], fabrication of electronic and photonic nanostructures [5, 7], [8], [9], failure analysis [10], and mask repair [11]. Ion implantation using a FIB has also been explored for fabrication of nanoscale devices such as quantum wires [12] and single electron transistors [13] in GaAs/AlGaAs, and Si p^+^-n junctions for CMOS [14] and CCD [15] applications. Compared to photolithography and e-beam lithography, FIB is a resist-free technique that enables direct etching or deposition of materials with lateral resolution comparable to e-beam lithography (i.e., on the scale of 10–100 nm) [5, 12, 13]. In this study, we advance the use of a commercial FIB system to locally modulate the optical properties of metal-oxide via doping or defect engineering. Previously, spatial control of doping [16–21] or defect density [22–28] has typically been accomplished by implanting ions from ion accelerators through lithographically defined masks, though the FIB has been used to locally tailor optical properties of Ge_2_Sb_2_Te_5_ (GST), a chalcogenide-based phase-change material [29, 30]. Here, we extend the use of the FIB to (a) modify the carrier concentration of zinc oxide (ZnO), a wide-bandgap semiconducting oxide, by area-selective doping, and (b) defect-engineer vanadium dioxide (VO_2_), a prototypical insulator-to-metal transition (IMT) material. The ability to tune the carrier density and phase-change behavior via focused ion-beam irradiation can enable local patterning of function in nanostructures.

## 2 Tunable carrier concentration in FIB-doped ZnO

The carrier concentration in most semiconductors can be tuned by orders of magnitude via *in situ* or *ex situ* doping processes, resulting in plasma wavelengths from the near infrared to the far infrared [16, 31], [32], [33], [34]. Doping can be performed *in situ* (i.e., during material growth) by tailoring the conditions to introduce dopants during growth processes such as sputtering [35], laser ablation [36], evaporation [37], chemical-vapor deposition [38]. In contrast, in *ex situ* doping techniques, dopants are introduced after material growth, for example via diffusion doping [39, 40], or ion implantation [41]. One advantage of ion implantation is that dopants can be introduced area-selectively, such as by implantation through lithographically defined masks, enabling designer structures, e.g., with plasmonic resonances. For example, we recently used this technique to locally tune the optical properties of silicon to realize all-silicon monolithic Fresnel zone plates and frequency-selective surfaces in the mid- and far-infrared [16]. In this section, we replace the conventional process of lithography and ion implantation with a FIB-based doping process, realizing mask-free area-selective doping.

We chose ZnO as the host material for FIB irradiation. Intrinsic ZnO is transparent from the visible to the mid-infrared, and can be *n*-type doped using gallium (Ga) [34], which is a common ion source in commercial FIB systems. Ga-doped ZnO has been demonstrated as a promising plasmonic material for infrared nanophotonics such as subwavelength waveguides [42–44], light-emitting diodes [45], and optical metasurfaces [46–48].

**Figure 1:**
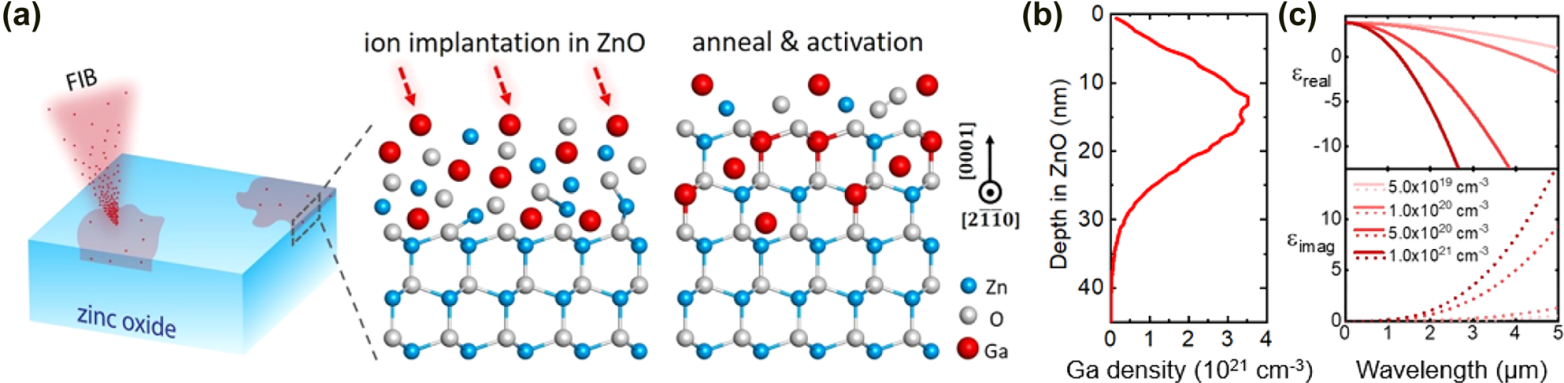
Mechanisms of tuning carrier density in ZnO using a focused ion beam (FIB). (a) Schematic of FIB-assisted doping process: The surface of a single-crystalline ZnO substrate can be doped using FIB implantation and subsequent high-temperature annealing. (b) Depth profile of 30-keV Ga ions impinging into crystalline ZnO, simulated using TRIM. (c) Calculated real and imaginary parts of the complex permittivity of Ga-doped ZnO with varying carrier concentrations.

The schematic of our FIB-assisted doping process is shown in Figure 1a: The ZnO wafer is bombarded by a 30-keV focused Ga ion beam, resulting in the implantation of Ga atoms into the top ∼30 nm of the ZnO lattice, but also resulting in lattice damage. A subsequent high-temperature annealing process is necessary for healing the damaged lattice and activating the dopants. As a result, an *n*-type Ga-doped ZnO layer is formed. The penetration depth profile of Ga ions into ZnO (Figure 1b) was estimated using a Monte-Carlo code, Transport of Ions in Matter (TRIM) [49], and verified in our samples using Auger electron spectroscopy (AES, Varian Inc.) and X-ray photoelectron spectroscopy (XPS; K-Alpha, Thermo Fisher Scientific) depth profiling (see Section 1 in Supporting Information).

The optical properties of metals and metal-like materials can often be approximated using the Drude model [50]. For Ga-doped ZnO, we anticipate that the Drude model should work well in the near-to-mid infrared, with the exception of wavelengths ∼20–25 µm, where there is a strong vibrational resonance that is intrinsic to ZnO [51]. In the Drude model, the complex permittivity $\left(\tilde{\varepsilon }\right)$ is given by

(1) $$\tilde{\varepsilon }\left(\omega \right)=\varepsilon_{\text{real}}+i\varepsilon_{\text{imag}}=\varepsilon_{\infty }\left(1-\frac{\omega_{p}^{2}}{\omega^{2}+i\frac{\omega }{\tau }}\right)$$

(2) $$\omega_{p}^{2}=\frac{n_{e}q^{2}}{m^{*}\varepsilon_{0}\varepsilon_{\infty }},\;\;\;\;\;\lambda_{p}=\frac{2\pi c}{\omega_{p}},\;\;\;\;\;\mu =\frac{\tau q}{m^{*}}$$

where *ɛ*_*∞*_ is the high-frequency permittivity, *ω*_*p*_ is the screened plasma frequency, which also corresponds to a plasma wavelength (*λ*_*p*_), *n*_*e*_ is the carrier concentration and *μ* is the carrier mobility determined by the scattering rate (*τ*), the effective mass of the free carriers (*m* ^*^), and the unit charge (*q*). The plasma wavelength is the wavelength at which the real part of the permittivity approaches zero, resulting in metal-like behavior at longer wavelengths. As shown in Figure 1c, we used the Drude model (Eqs. (1) and (2)) to calculate the complex permittivity $\left(\tilde{\varepsilon }\right)$ of the Ga-doped ZnO for carrier concentrations from 5 × 10^19^ to 1 × 10^21^ cm^−3^, in which the plasma wavelength is blue-shifted toward the near infrared as the carrier concentration increases.

We irradiated several single-crystalline (0001) ZnO substrates (10 × 10 mm^2^, CrysTec GmbH) with 30-keV Ga ions at room temperature using a commercial FIB system (FEI 600i nanoLab). On each sample, five 200-by-200 µm areas were homogenously implanted with ion fluences of 3.6 × 10^14^, 6 × 10^14^, 1.2 × 10^15^, 3.6 × 10^15^, and 6 × 10^15^ cm^−2^ (corresponding to Ga peak concentrations of 0.31, 0.52, 1, 3.1, and 5.2 at.%, respectively), which are close to and above the solid solubility limit of Ga in ZnO [52–54]. Note that we irradiated 200-by-200 µm areas to enable far-field optical characterization; in principle, nanometer-scale (10–100 nm) lateral resolution can be achieved for the implantation process in a commercial FIB system if diffusion can be avoided. To heal the damaged lattice and activate the Ga dopants, we then performed 40-minute thermal annealing treatments in air of the irradiated samples. Each sample was annealed at a different temperature ranging from 600 to 1000 °C, respectively (complete data and plots for all annealing temperatures can be found in the Supporting Information Section 2).

**Figure 2:**
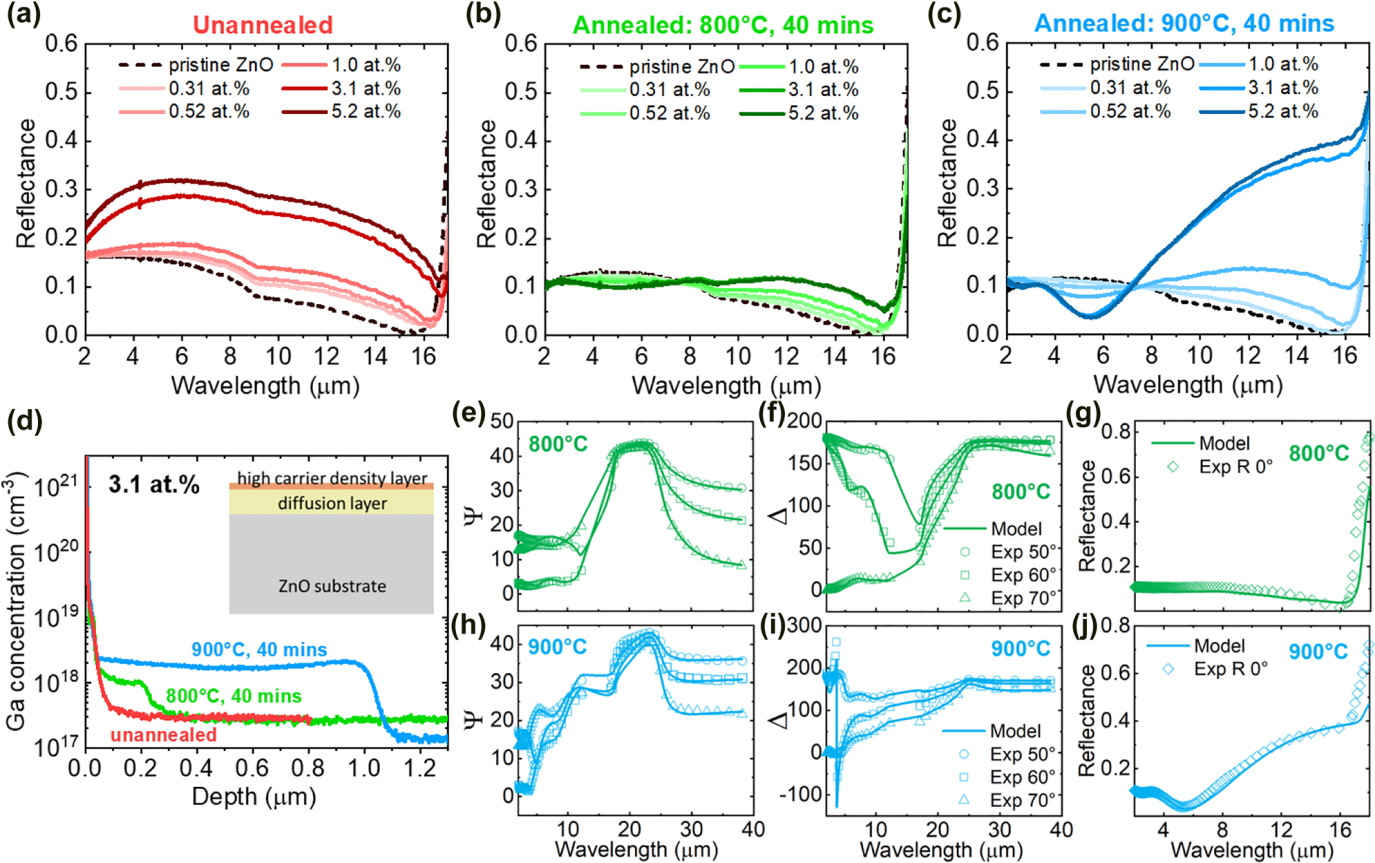
Measured normal-incidence reflectance for undoped ZnO, and FIB-irradiated ZnO regions with different ion fluences. (a) without annealing treatment, (b) followed by annealing in air at 800 °C and (c) 900 °C, respectively, for 40 min. (d) SIMS depth profiles of the Ga concentration in the samples implanted using an ion accelerator (not a FIB, to enable very large area implantation for infrared ellipsometry and SIMS). The ion energy was identically 30 keV and the peak doping concentration was chosen to be 3.1 at.%. The inset is the schematic showing the three-layer depth profile of thermal annealed FIB-ZnO. (e-j) The experimental (discrete points) and model fitted (solid curves) ellipsometric parameters (Ψ and Δ) and normal-incidence reflectance for the 3.1 at.% sample annealed at 800 °C (e-g) and for the other one annealed at 900 °C (h-j).

To study the changes in optical properties caused by various doping concentrations and annealing treatments at different temperatures, we performed reflectance measurements on each of these FIB-ZnO regions using a Fourier-transform infrared (FTIR) spectrometer (Bruker Vertex 70) outfitted with an infrared microscope (Hyperion 2000). For the unannealed Ga:ZnO samples (Figure 2a), we observed increasing reflectance with respect to increasing Ga ion fluence, which we attribute to the partial activation of Ga dopants even without an annealing treatment. Our assumption is supported by a comparison with ZnO substrates that were implanted with Kr ions (see details in Supporting Information Section 3).

As observed in Figure 2b and c, the increase of the reflectance versus doping concentration at longer wavelengths (>8 µm) is as expected due to the activation of dopants. The reduction of the reflectance at shorter wavelengths is likely due to the diffusion of Ga during the annealing treatments which can result in µm-thick doped layers, causing Fabry–Pérot (F-P) fringes at shorter wavelengths (<8 µm).

To quantitatively extract physical properties such as carrier concentration and mobility, we performed spectroscopic ellipsometry analysis, which requires centimeter-scale irradiation areas. Therefore, we prepared another set of ZnO substrates irradiated by comparable ion fluences and identical ion energy of 30 keV using an ion implanter, enabling us to homogeneously implant an entire 1-by-1 cm ZnO substrate. Then, we performed spectroscopic ellipsometry (IR-VASE Mark II, J. A. Woollam Co.) measurements for wavelengths from 2 to 20 µm and built a model using ellipsometry analysis software (WVASE, J. A. Woollam Co.) to fit the data. Our assumption about diffusion was confirmed by secondary ion mass spectrometry (SIMS, implemented by Qspec Technology, Inc.) depth profiles as shown in Figure 2d. We found ∼0.2 and ∼1 µm plateaus in the 3.1 at.% samples annealed at 800 °C and 900 °C, respectively, which are clear evidence of the diffusion of Ga dopants. Therefore, we built a three-layer model, consisting of a semi-infinite single-crystalline ZnO substrate, one diffusion layer with low carrier concentration, and one top-surface layer with high carrier concentration (inset in Figure 2d). In our model, we first characterized the pristine ZnO substrate using seven Gaussian oscillators, and for the Ga-doped ZnO (both the top-surface layer and the diffusion layer), an additional Drude oscillator function was added into the oscillator functions of pristine ZnO, to account for the induced carrier concentration due to the doping (see details in Supporting Information Section 4). Therefore, the fitting parameters for each sample were the thicknesses, carrier concentrations, and mobilities for the two layers. Note that we kept the seven Gaussian oscillators fixed and only the Drude term was fitted. We used prior knowledge about the thickness of the diffusion layer from SIMS data (Figure 2d) to constrain the fitting for just that parameter; specifically, we constrained the diffusion-layer thickness from 180 to 250 nm for the 800 °C annealed sample, and 0.9–1.1 µm for the 900 °C annealed sample.

As shown in Figure 2e–j, our model fitted well with the experimental data (Ψ and Δ) acquired using spectroscopic ellipsometry. Note that we excluded the data between 12 and 17 µm in the fitting to avoid non-physical spikes in Ψ and Δ, which result from the low reflectivity of our samples within that wavelength range (more discussion can be found in Supporting Information Section 5). The carrier concentration of the 3.1-at.% samples annealed at 800 °C and 900 °C reached 10^20^ cm^−3^ in the top-surface layer, while the carrier concentrations in the diffusion layers underneath are two orders of magnitude lower (Table 1). These fitting results agreed with our SIMS characterizations that the Ga dopants were diffusing from the implantation profile during the annealing process, resulting in a much thicker diffusion layer with a much lower carrier concentration. For most applications, such diffusion layers are unwanted since they trade off patterning resolutions and optical contrast between FIB-irradiated and pristine regions. Since the diffusion layer is highly correlated to the annealing conditions (i.e., annealing temperature and annealing time), plausible methods to decrease the annealing time such as flash lamp annealing [55, 56] and laser annealing [57–59] could be useful for suppressing the diffusion.

**Table 1:** Drude-fitting parameters of the two 3.1 at.% samples annealed at 800 °C and 900 °C, respectively.

3.1 at.% Ga:ZnO	High carrier concentration layer			Diffusion layer		
	Thickness, nm	*n*_*e*_, cm^−3^	μ, cm^2^/V·s	Thickness, nm	*n*_*e*_, cm^−3^	μ, cm^2^/V·s
800 °C	8.0	1.25 × 10^20^	19.94	205.4	1.23 × 10^18^	316.47
900 °C	8.0	2.16 × 10^20^	17.80	1008.3	2.40 × 10^18^	232.59

## 3 Tunable phase-transition characteristics in FIB-engineered VO_2_

In the previous section, we demonstrated that optical properties such as carrier density and mobility of an oxide semiconductor (here, ZnO) can be locally modified via a simple step of mask-free FIB-assisted ion implantation, followed by a thermal annealing process. We can also use FIB implantation (without annealing) to intentionally introduce structural defects into a material, and the induced defect density can be continuously controlled by varying the ion fluence. Such defect-engineering techniques can be useful for modulating physical properties of materials, especially for strongly correlated electron systems in which electronic properties are very sensitive to changes in the lattice parameters [22, 60–72]. In this section, we show that such FIB-assisted defect-engineering can be used to locally modulate the IMT temperature of thin-film VO_2_, an electron-correlated material that undergoes an IMT at ∼70 °C [73, 74] and features an orders-of-magnitude change in carrier density. The IMT temperature of VO_2_ is determined by the stability of the electron hybridization, which is very sensitive to the strain environment in the thin film [22, 23, 75–77]. We previously demonstrated that the IMT temperature can be tuned by introducing structural defects in the VO_2_ film via high-energy ion irradiation performed using an ion accelerator, where we found the change in optical properties and IMT temperature of VO_2_ depend on the density of generated defects, but not on the particular ion species (Ar or Cs), and the generated defects introduce more strain to the surrounding and lower the IMT temperature [22].

**Figure 3:**
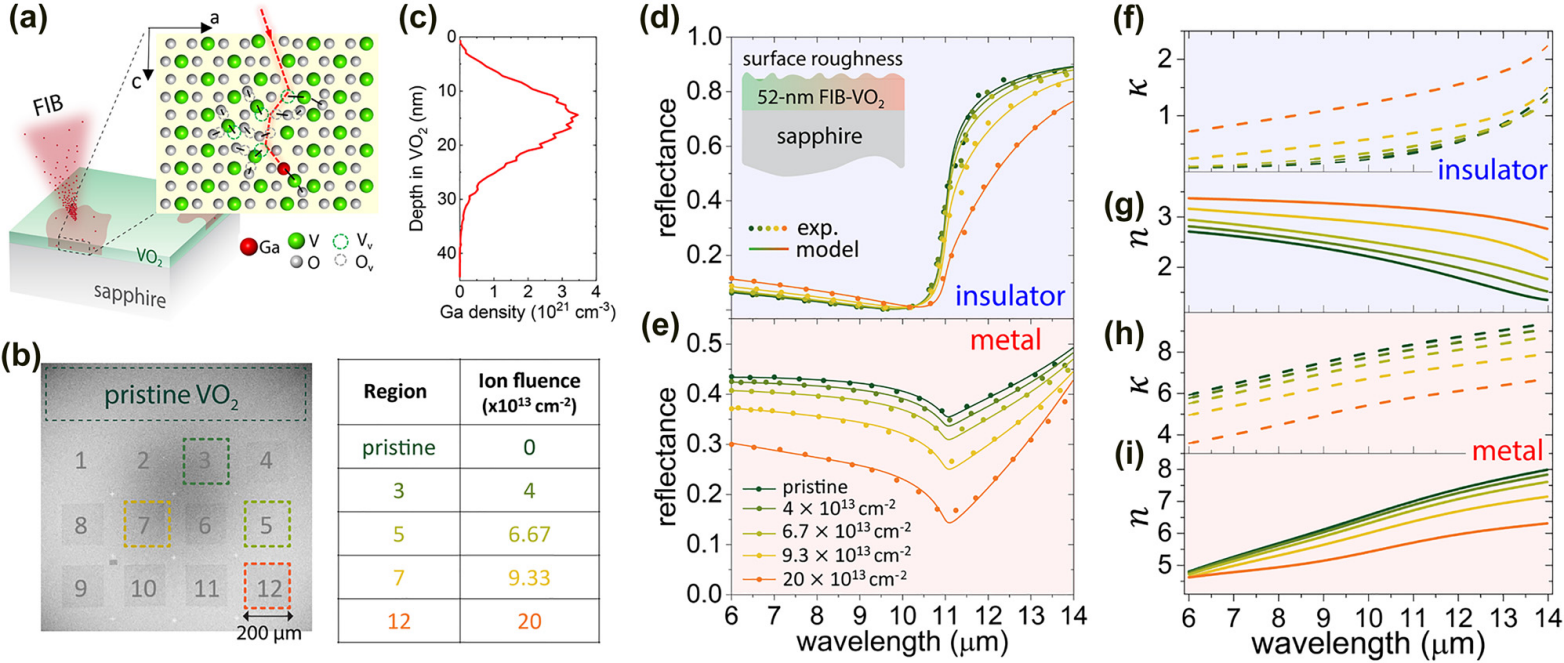
FIB-induced changes in the optical refractive index of VO_2_. (a) Ion irradiation of VO_2_ using a FIB system, with an inset schematic of the defect-engineering process, showing a collision cascade in the VO_2_ lattice initiated by an energetic Ga ion. (b) SEM image of the FIB-irradiated VO_2_ regions with the corresponding ion fluences listed. (c) Simulated depth profile of 30-keV Ga ions into a VO_2_ thin film using TRIM. (d, e) The symbols are FTIR reflectance measurements on pristine VO_2_ and regions irradiated with ion fluences of 4 × 10^13^, 6.7 × 10^13^, 9.3 × 10^13^, 20 × 10^13^ cm^−2^, for temperatures at 25 °C (all regions in pure insulating phases) and 100 °C (all regions in pure metallic phases), respectively. The solid curves are the model fits to the FTIR measurements, where the underlying model was created based on ellipsometry of pristine VO_2_. The insulator-phase (f, g) and metal-phase (h, i) refractive indices were extracted from the fittings shown in (d) and (e).

Here, we show that high-resolution mask-free defect engineering can be accomplished using a commercial FIB system. Similar to the FIB irradiation of ZnO (before annealing), here structural defects are introduced by the collision cascades of impinging ions and lattice atoms (V and O) (Figure 3a), causing changes in the strain environment in the film and thus the IMT temperature is expected to be modulated to different extent depending on the ion fluence.

We deposited a ∼50-nm VO_2_ film on *c*-plane sapphire via magnetron sputtering [74, 78]. Then, twelve 200-by-200 µm regions were irradiated using focused 30-keV Ga ions at room temperature with varying ion fluences up to 2 × 10^14^ cm^−2^, as shown in Figure 3b. The density of induced structural defects is proportional to the density of Ga ions implanted into the VO_2_ film, which we estimated using TRIM simulations (Figure 3c).

To investigate the irradiation-induced changes in the optical properties of the pure insulator- and metal-phase VO_2_, we first performed reflectance measurements on each of these FIB-irradiated VO_2_ regions using our FTIR spectrometer with microscope, for temperatures of 25 °C (i.e., VO_2_ in the pure insulating phase for all the irradiated regions) and 100 °C (i.e., VO_2_ in the pure metallic phase for all the irradiated regions), as shown in Figure 3d and e. Then, we fitted the measured reflectance by adjusting the parameters of a model that we previously built to characterize refractive indices of intrinsic thin-film VO_2_ [74]. As shown in the inset of Figure 3d, the model consisted of a semi-infinite anisotropic *c*-plane sapphire [74], a VO_2_ layer, and surface roughness (50% air + 50% of the material underneath). For the insulating phase, the dielectric function of the VO_2_ layer is a series of Lorentzian oscillators. For the metallic phase, we also used Drude functions to capture the contribution of the free carriers (more details can be found in ref. [74]). The thicknesses of VO_2_ and surface roughness were set to 52 and 5 nm, respectively, based on SEM imaging of the cross section (Supporting Information Section 6). We were able to fit our reflectance measurements (Figure 3d and e) by only adjusting the line shapes, amplitudes, and spectral positions of the Lorentz and Drude functions. Therefore, the complex refractive indices of VO_2_ for different ion fluences can be extracted, as plotted in Figure 3f-i.

**Figure 4:**
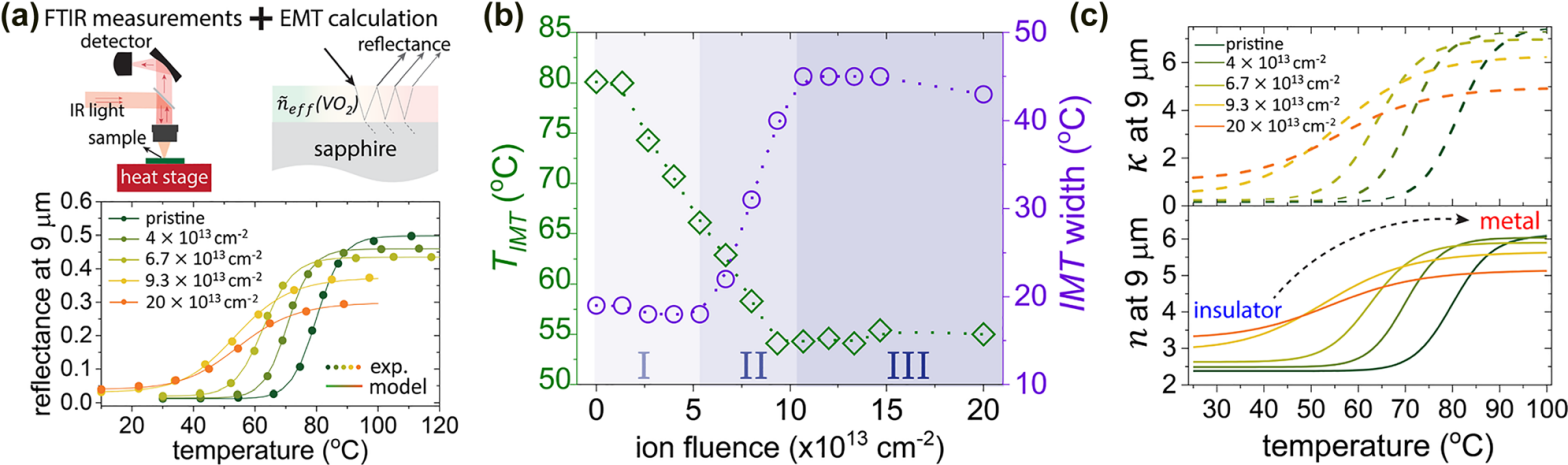
FIB-induced changes in phase-transition temperature and width of VO_2_. (a) Temperature-dependent optical characterization of the FIB-irradiated VO_2_. First, we measured temperature-dependent reflectance across the IMT for each irradiated region. Then, we applied effective-medium theory to approximate the refractive indices at intermediate temperatures and calculate the temperature-dependent reflectance. By sweeping the parameters of *T*_IMT_ and *E*—which determine the IMT width and temperature, respectively—in Eq. (4), we found the best fit between the FTIR measurements and calculation, enabling us to extract the IMT temperature and width for each irradiation ion fluence, as plotted in (b). (c) Extracted temperature-dependent refractive indices of the defect-engineered VO_2_ irradiated by different ion fluences. Here we plot the results for a single wavelength of 9 µm to clearly show the evolution of refractive-index values versus temperature and ion fluence.

Then, we investigated FIB-induced modulation of the IMT temperature and width by a combination of temperature-dependent FTIR reflectance measurements and effective-medium theory, as schematically shown in Figure 4a. FTIR reflectance measurements were performed on all irradiated regions for temperatures increasing from 10 to 120 °C, in steps of 2 °C. We observed that the phase transition shifted to lower temperatures as the ion fluence increased, which agrees with our previous observations for defect-engineered VO_2_ irradiated using an ion accelerator [22]. To quantitatively study the changes of IMT characteristics with respect to the FIB fluence, we used the Looyenga effective-medium theory formalism [59] to approximate the refractive indices of the irradiated VO_2_ at intermediate temperatures [74, 79]:

(3) $${\tilde{\varepsilon }}_{\text{eff}}^{1/3}=\left(1-f\right){\tilde{\varepsilon }}_{i}^{1/3}+f{\tilde{\varepsilon }}_{m}^{1/3}$$

where $\tilde{\varepsilon }={\tilde{n}}^{2}={\left(n+i\kappa \right)}^{2}$ is the complex dielectric function of VO_2_ and *f* is the temperature-dependent volume fraction of the metal-phase VO_2_ domains within the film. The co-existence of insulating and metallic domains can be understood as a first-order equilibrium distribution, and therefore *f*(*T*) can be expressed as [22, 80]:

(4) $$f\left(T\right)=\frac{1}{1+\exp\left[E/k_{B}\left(1/T-1T_{IMT}\right)\right]}$$

where *E* is an energy scale that determines the sharpness of the IMT (i.e., inversely proportional to the IMT width). *T*_IMT_ is the temperature where 50% of VO_2_ transformed to the metallic phase in a heating process.

For given *E* and *T*_IMT_, we used Eqs. (3) and (4) to obtain the temperature-dependent refractive indices and then calculated the optical reflectance of each irradiated region using the transfer-matrix method. As shown in Figure 4a, by sweeping *E* and *T*_IMT_, we achieved good agreement between the calculation (solid curves) and FTIR measurements (dotted lines). The fitted IMT temperature and width as a function of ion fluence are shown in Figure 4b. The IMT width is defined to be the temperature interval between where 3% of VO_2_ is in the metallic phase and where 97% of VO_2_ is in the metallic phase. Note that due to the hysteresis in VO_2_, the value of *T*_IMT_ is different for heating and cooling [74, 81]. Once $f\left(T\right)$ was determined, we were able to obtain the temperature-dependent refractive indices across the IMT for each irradiated region, as shown in Figure 4c. Here, we only plot the results for a single wavelength (*λ* = 9 µm) to better show the evolution of the refractive indices with respect to both the temperature and the FIB fluence. The full dataset for wavelengths from 6 to 14 µm can be found in Supporting Information Section 7.

As shown in Figure 4b *,* there are three distinct ion fluence regimes (labeled in the figure as I, II, and III), in which the IMT characteristics evolve differently. For ion fluences <5 × 10^13^ cm^−2^, the IMT temperature gradually decreases with fluence, with a reduction of ∼15 °C for 5 × 10^13^ cm^−2^ and no substantial changes in either the refractive index of the two pure phases or in the IMT width. At higher ion fluences between 5 × 10^13^ and 1.1 × 10^14^ cm^−2^, we observed that the IMT temperature could be further shifted to lower temperatures, but the shift was accompanied by a significant broadening in the IMT width and a reduction in the refractive-index contrast between the two pure phases (Figure 4c).

We attribute such distinct phenomena to the different defect morphologies induced by different levels of ion fluence. At low ion fluences, the impinging ions mostly cause point defects that can reduce the transition temperature due to local compressive strain [22, 77]. Such point defects are in much smaller than the probing wavelengths of our FTIR measurements. The strain induced by the point defects can be redistributed and partially relaxed at room temperature after irradiation [82, 83], resulting in a homogeneous strain environment in the film. This understanding is consistent with the lack of broadening in the IMT width in the low-fluence irradiation regions in Figure 4. As the ion influence increases, point defects are expected to accumulate and form nanometer-sized defect complexes that can affect the IMT temperature in microscopic scales, thus resulting in apparent broadening of the IMT in regions irradiated with high fluence.

When the ion fluence surpasses ∼1.1 × 10^14^ cm^−2^, both the IMT temperature and width became constant versus the increasing ion fluence, likely due to the limited penetration depth of the 30-keV Ga ions in the VO_2_ film. At these high ion fluences, we expect the density of the induced structural defects complexes to saturate (i.e., complete amorphization occurs [22]) within the depth of ∼30 nm from the VO_2_ surface, while leaving a less-affected VO_2_ layer underneath, as shown in our TRIM simulation (Figure 3c).

## 4 Summary

We have shown that the optical properties of two oxide materials, zinc oxide (ZnO) and vanadium dioxide (VO_2_), can be locally modulated by doping or defect engineering using a commercial focused ion beam (FIB) with gallium ions. Using the FIB, we modified the carrier concentrations in initially undoped ZnO, reaching carrier concentrations as high as 10^20^ cm^−3^, and reduced the temperature of the insulator-to-metal transition (IMT) in VO_2_ by as much as ∼25 °C. The FIB process does not require any lithography or masking, and only requires one additional annealing step in the case of doping. Due to the versatility of commercial FIBs, this technique can be used to modify and engineer materials with high resolution even for the case of irregularly shaped materials where conventional lithography is challenging. The ability to dope and defect-engineer certain oxides using a commercial FIB provides functionalities beyond the more-common FIB milling and deposition, and may enable the direct fabrication of a broader range of infrared devices based on semiconducting oxides.

## Supplementary Material

Supplementary Material Details

## Data Availability

The data that support the findings of this study are available from the corresponding author upon reasonable request.
