# Supplementary material for: Tuning carrier density and phase transitions in oxide semiconductors using focused ion beams
Source: Nanophotonics. 2022 Jun 13;11(17):3923–32. doi: 10.1515/nanoph-2022-0050 (PMC11501530; doi:10.1515/nanoph-2022-0050)
Supplement: Supplementary file 1 — Supplementary Material Details [file j_nanoph-2022-0050_suppl.docx]

**Supporting Information:**

**Tuning carrier density and phase transitions in oxide semiconductors using focused ion beams**

Hongyan Mei^1*^, Alexander Koch^2*^, Chenghao Wan^1,3*^, Jura Rensberg^2^, Zhen Zhang^4^, Jad Salman^1^, Martin Hafermann^2^, Maximilian Schaal^2^, Yuzhe Xiao^1^, Raymond Wambold^1^, Shriram Ramanathan^4^, Carsten Ronning^2†^, Mikhail A. Kats^1,3,5†^

*^1^ Department of Electrical and Computer Engineering, University of Wisconsin-Madison, Madison, Wisconsin 53706, USA*

*^2^ Institute of Solid State Physics, Friedrich-Schiller-Universität Jena, Jena, Thuringia 07743, Germany*

*^3^ Department of Materials Science and Engineering, University of Wisconsin-Madison, Madison, Wisconsin 53706, USA*

*^4^ School of Materials Engineering, Purdue University, West Lafayette, IN 47907, USA*

*^5^ Department of Physics, University of Wisconsin-Madison, Madison, Wisconsin 53706, USA*

**These authors contributed equally to this work.*

*^†^E-mail:* [*mkats@wisc.edu*](mailto:mkats@wisc.edu) *,* [*Carsten.Ronning@uni-jena.de*](mailto:Carsten.Ronning@uni-jena.de)

**Section 1. X-Ray Photoelectron Spectroscopy (XPS) and Auger Electron Spectroscopy (AES) of Ga:ZnO**

**X-Ray Photoelectron Spectroscopy (XPS)**

XPS depth profiling (K-Alpha, Thermo Fisher Scientific) was performed to investigate the chemical binding states and the relative atomic composition in Ga:ZnO. Four samples were investigated: intrinsic zinc oxide (ZnO), as-implanted Ga:ZnO (i.e., samples without annealing treatments) and two Ga:ZnO samples annealed at 900 °C and 1000 °C for 40 minutes. All Ga:ZnO samples were implanted with a ion fluence of 6 × 10^15^ cm^-2^, which corresponds to Ga peak concentration of 5.2 at.% at an ion range of ~14 nm and a straggling of ~6 nm, which was calculated using an open-source Monte Carlo code, Transport of Ions in Matter (TRIM) [S1].

First, we performed a survey scan on the as-implanted Ga:ZnO to estimate surface contaminations and detectable elements. Then, we performed highly resolved XPS measurements on each detectable element. The Shirley background subtraction and the peak fitting with asymmetric Lorentzian or Gaussian line shape profiles were executed on the high-resolution XPS spectra for chemical state analysis (implemented in the CasaXPS [S3]). We report XPS depth profiles of Ga2p, Zn2p, O1s and C1s. The depth profiles were realized with a stepwise etching process using Ar ions with an etching rate of 0.1 nm/s. We first removed the surface contaminations with an 50-second etching and then started with a depth profiling of Ga:ZnO for 600 seconds in total (etching for 10 seconds per cycle, 31 scans in total) to get depth resolved XPS spectra including both the Ga-implanted and non-implanted region.

The survey scan of the as-implanted Ga:ZnO surface is shown in Fig. S1, in which peaks of Zn, O, C and Ga were detected. The spectrum shows nontrivial surface contamination with C and O due to the long exposure in air.


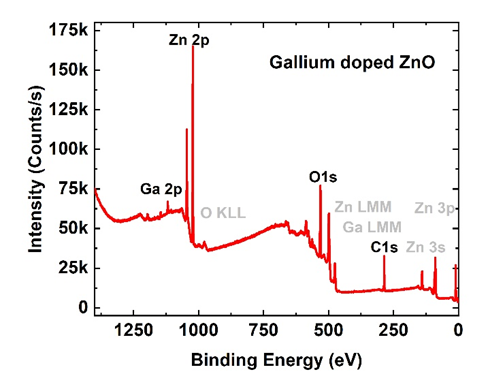


**Figure S1.** XPS survey scan of as-implanted Ga:ZnO sample surface with binding energies for several peaks.

Fig. S2(a) shows the highly resolved C1s spectra with three components, which describes most probably adventitious carbon contamination on the Ga:ZnO surface [S2]–[S4]. The main peak I (285.7 eV) represents pure carbon (C-C), peak II (287 eV) stands for carboxyl groups (C-O) and peak III (289.7 eV) describes carbonates (O-C=O) [S2]–[S4]. All Ga:ZnO samples exhibit a similar carbon contamination at the surface, which need to be removed by Ar^+^ etching for element quantification of Ga:ZnO. Fig. S2(a) includes the peak intensity of C1s in function of etching depth. In total we etched for 600 s, i.e., 60-nm deep from surface into the substrate. After 50 seconds of etching we could still see a small peak for pure carbon (at the location of the blued dashed line), which was very likely due to the recoil implantation.

After 50 seconds of etching, we started depth profiling for the element quantification and chemical binding state analysis, in which the Zn2p- [Fig. S2(b)], O1s- [Fig. S2(c)] and Ga2p- [Fig. S2(d)] peaks were considered. As shown in Fig. S2(b) the peak composition (i.e., the binding state) of Zn2p does not change with respect to the depth. The binding energies of 1022.5 eV and 1045.5 eV are corresponding to Zn2p_3/2_ and Zn2p_1/2_ within the tetrahedral coordination of crystalline ZnO [S2], [S3], [S5], [S6]. The Zn2p_3/2_ peak shifted from 1022.5 eV to 1022.2 eV during 600 seconds of etching, indicating a small portion of Zn-O bonds were either broken or substituted by Ga-O bonds after the Ga ion implantation.


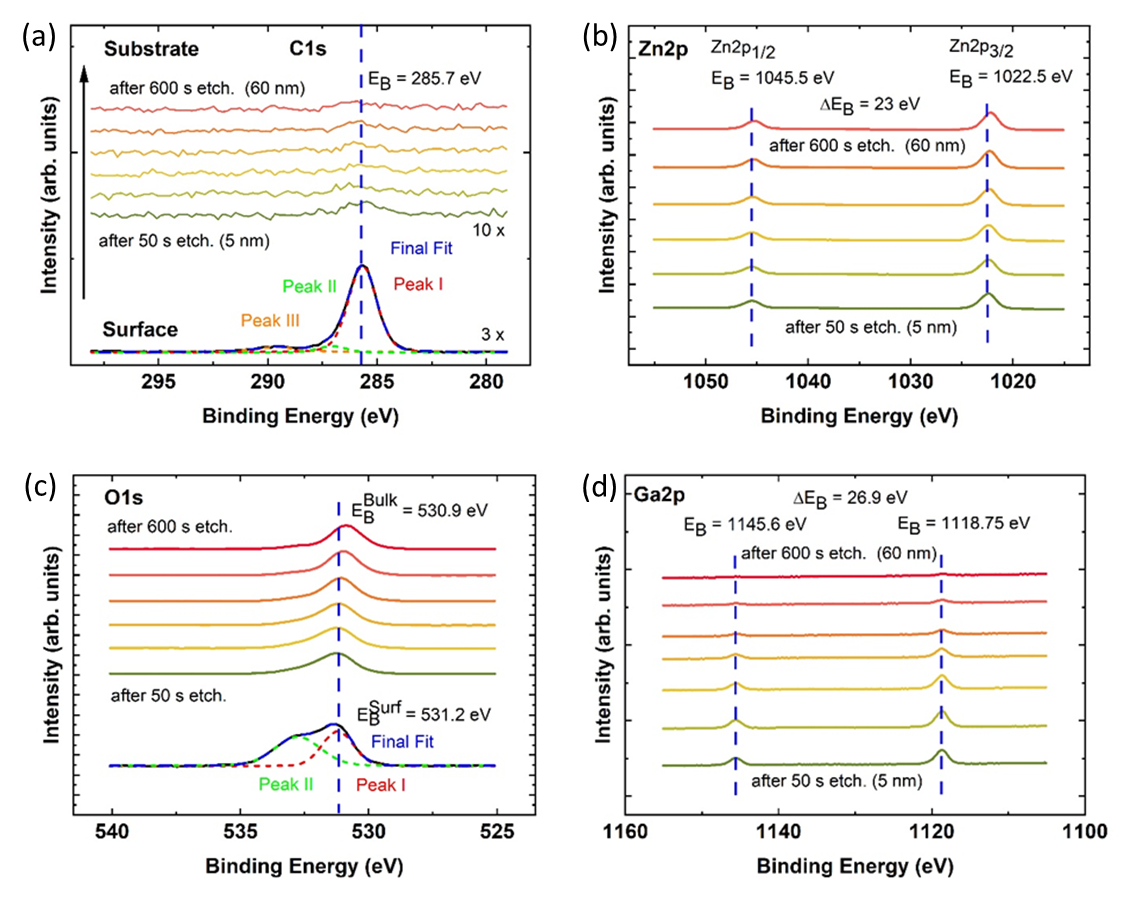


**Figure S2. (a)** Surface and depth profile of C1s spectra of the as-implanted Ga:ZnO sample. The depth profile (green-to-red lines) is a series of highly resolved XPS spectra measured in a sequence of 100-second etchings. For a better view the C1s spectra are scaled up. **(b)** Depth profile of Zn2p spectra of the unannealed Ga:ZnO substrate. **(c)** Surface and depth profile of O1s spectra for the as-implanted Ga:ZnO. **(d)** Depth profile of Ga2p spectra of the as-implanted Ga:ZnO.

As shown by the solid blue curve in Fig. S2(c), the surface XPS spectra of the O1s can be fitted by two Gaussian peaks: peak I at 531.2 eV that is attributed to Zn-O bonding and peak II at 532.7 eV that indicates the presence of O-H bonds induced by the surface contamination [S2], [S3], [S5], [S6]. After the initial 50-second etching, Peak II disappeared because the surface contamination was removed. Then, within the further etching, Peak I shifted by ~0.3 eV, similar as that of the Zn2p_3/2_ peak, which is due to the lattice damage during the Ga ion implantation.

In the depth profile of the Ga2p spectra [Fig. S2(d)], the bonding energies are found to be 1118.75 eV for Ga2p_3/2_ and 1145.6 eV for Ga2p_1/2_, which are 2 eV higher than the binding energies in Ga metal, indicating a binding between Ga and O in the ZnO substrate [S1], [S2], [S4]–[S6]. On the other hand, there is no peak at 1117.5 eV [S2], [S7], which is the typical bonding energy of Ga-O in gallium oxide(Ga_2_O_3_), indicating no macroscopic agglomerates of Ga_2_O_3_ formed during the implantation or the concentration of Ga_2_O_3_ is less than the XPS-detection limit. In addition, Ga2p peak intensity is decreasing as a function of depth, which agrees well with our TRIM simulation results shown in Fig. 1(b) in the main text.

Fig. S3 plots the concentration of Ga as a function of depth for the as-implanted Ga:ZnO and the samples annealed at 900 °C and 1000 °C. In general, the Ga concentration in the annealed samples were found to be lower than that of the as-implanted sample, indicating the thermal diffusion of Ga from the surface into the bulk ZnO during the annealing process. Furthermore, such diffusion is highly dependent on the annealing temperature, as we found that the Ga concentration left at the surface region in the sample annealed at 1000 °C (blue curve) is much less than that of the sample annealed at 900 °C (red curve). We also observed several subpeaks of Ga concentration in the annealed samples (two in the red curves and one in the blue curve), indicating a re-accumulation process of Ga that is also depending on annealing conditions (i.e., temperature and time). Though the dynamic of such re-accumulation has not been fully understood, we believe it can be avoided by a more-sufficient annealing treatment (i.e., annealing with either sufficiently high temperature or sufficiently long time).


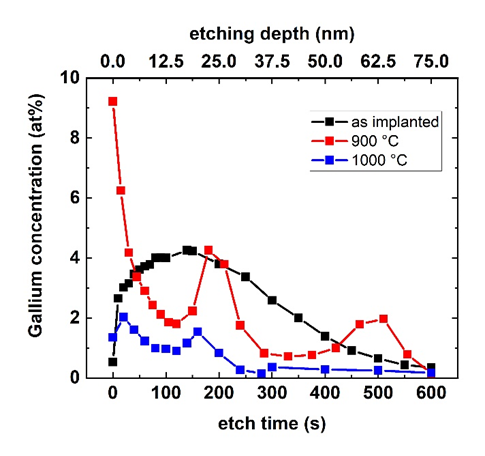


**Figure S3.** Depth profiles of Ga concentration for the as-implanted Ga:ZnO and Ga:ZnO samples annealed at 900 °C and 1000 °C for 40 minutes.

**Auger Electron Spectroscopy (AES)**

We also performed AES measurements (Varian Inc.) on the same as-implanted Ga:ZnO to confirm the depth profile of the Ga concentration, by comparing the results to those of the TRIM simulation and the XPS measurements. The depth profiling was done via a stepwise etching process using krypton (Kr). The total etching process resulted in a removal of 68-nm material. The etching rate was 0.12 nm/s with the step size of 30 seconds.


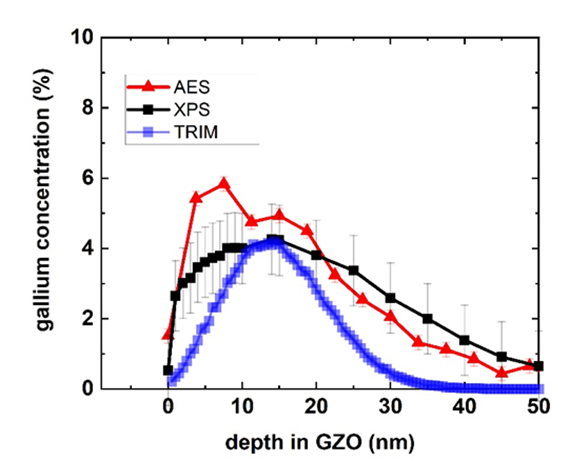


**Figure S4.** Comparison between AES, XPS, and TRIM results of the depth profiling of Ga concentration in the as-implanted Ga:ZnO sample that was implanted by 30-keV Ga ions with a fluence of 6×10^15^ cm^-2^.

Comparison among the results of AES, XPS, and TRIM simulation is summarized in Fig. S4. As discussed in the main text, TRIM simulation predicts a Gaussian-like depth profile of the Ga distribution with the peak concentration, c_Ga_, of ~5.2 at.%, which matches well with that of the XPS measurements. The measured depth profiles by XPS or AES featured broader Ga distribution ranges than that of the TRIM simulation which is probably due to slight thermal diffusion of Ga caused by the generated heat during the implantation.

We estimated the total amount of implanted Ga by calculating area under the concentration curves. For the TRIM simulation, the total implanted Ga concentration, c_Ga,_ is 6 × 10^22^ cm^-3^. For the experiments, we got c_Ga_ of 1.23 × 10^23^ cm^-3^ from AES results and 1.18 × 10^23^ cm^-3^ from XPS results, which agree well with each other.

**Section 2. FTIR reflectance of Ga:ZnO samples annealed at different temperatures**

Several single-crystalline ZnO substrates were irradiated using a 30-keV focused Ga ion beam at room temperature. On each sample, we homogenously implanted five 200-by-200-µm areas with different ion fluences that correspond to Ga concentrations of 0.31, 0.52, 1, 3.1, and 5.2 at.%. Then, the samples were thermally annealed in air for 40 minutes at temperatures of 600, 700, 800, 900 and 1000 °C. Optical reflectance of these FIB-ZnO regions were measured using our FITR microscope and the results are plotted in Fig. S5, in which three major trends were found: a) For the unannealed samples [Fig. S5(a)], the increase of the reflectance with respect to the increasing ion fluence is likely due to the introduction of Ga ions and structural defects at the ZnO surface. After the annealing at lower temperatures [i.e., Fig. S5(b and c)], the reflection magnitudes and line shapes of those FIB-ZnO regions “recovered” to that of the undoped ZnO spectrum, which could be due to multiple effects including healing of the induced defects, the formation of Ga-O bonds, and the diffusion of Ga ions into the bulk ZnO. b) In Fig. S5(d, e, and f), we observed an obvious increase in the reflectance with respect to the increasing Ga concentration at longer wavelengths (> 8 µm), which is attributed to the activation of dopants during the annealing process. c) On the other hand, at shorter wavelengths (< 8 µm) the reduction of reflectance is likely due to the diffusion of Ga during the annealing treatments, resulting in µm-thick less-doped layers that caused Fabry–Pérot fringes.

**
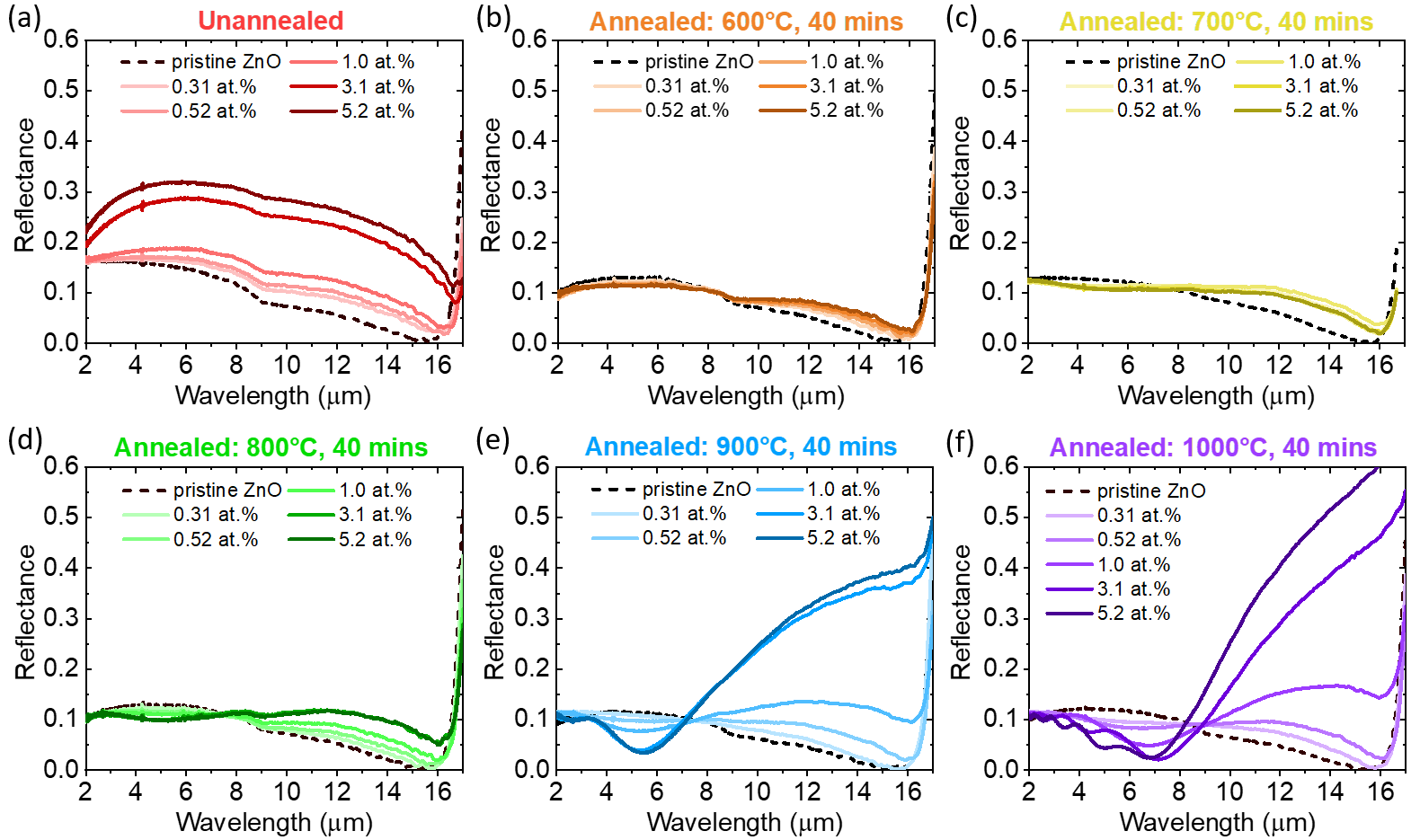
**

**Figure S5.** Measured near-normal-incidence reflectance for undoped ZnO, and areas irradiated with different ion fluences followed by **(a)** no annealing treatment, **(b-f)** annealing in air for 40 minutes at temperatures of 600, 700, 800, 900, and 1000 °C, respectively.

**Section 3. Comparison between ZnO implanted by Kr and Ga ions**

In order to understand the increasing reflectance with the increasing Ga ion fluences in the unannealed samples, we implanted ZnO with 30-keV Krypton (Kr) ions using an ion implanter, for ion fluence ranging from 1.6$\times$10^15^ cm^-2^ to 1.6 $\times$10^16^ cm^-2^, resulting in similar defect concentration distribution as that of Ga ions. Kr is a noble gas with an atomic mass of 84, which is slightly heavier than Ga with an atomic mass of 69. Therefore, the ion fluences we used for Kr is a little bit smaller than for Ga. Figure S6(a) shows the generated vacancy profiles at 30 keV versus the target depth, according to TRIM calculations [S1].


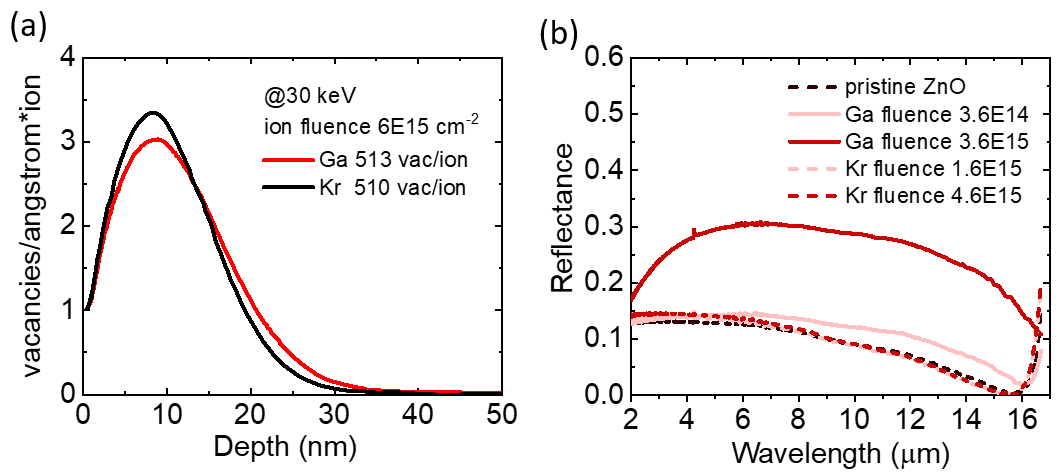


**Figure S6.** **(a)** TRIM-calculated vacancy distribution of Ga and Kr implantation at 30 keV, both with an ion fluence of 6 × 10^15^ cm^-2^. **(b)** Reflectance of Kr-implanted single-crystalline ZnO and Ga-implanted single-crystalline ZnO, both without annealing.

Figure S6(b) presents the FTIR reflectance of ZnO implanted with Kr and Ga ions using similar implantation conditions. The reflectance of the Kr-implanted ZnO is close to that of the pristine ZnO, indicating that any implantation-induced defects do not affect the optical properties. In contrast, the reflectance of ZnO significantly increased after the Ga implantation, which we attributed to some Ga dopant activation even without annealing, resulting in a thin layer of highly doped Ga:ZnO.

**Section 4. Surface morphology of Ga:ZnO samples annealed at different temperatures**

We took SEM images of the FIB-ZnO areas irradiated with the fluence of 6×10^15^ cm^-2^ and annealed at different temperatures (Fig. 5). For the samples annealed at high temperatures [Fig. S5(c and d)], the process of the recrystallization was observed. The hexagonal features in Fig. 5(d) are likely the wurtzite ZnO [S8], which is the most thermally stable form for single crystalline ZnO in ambient air.


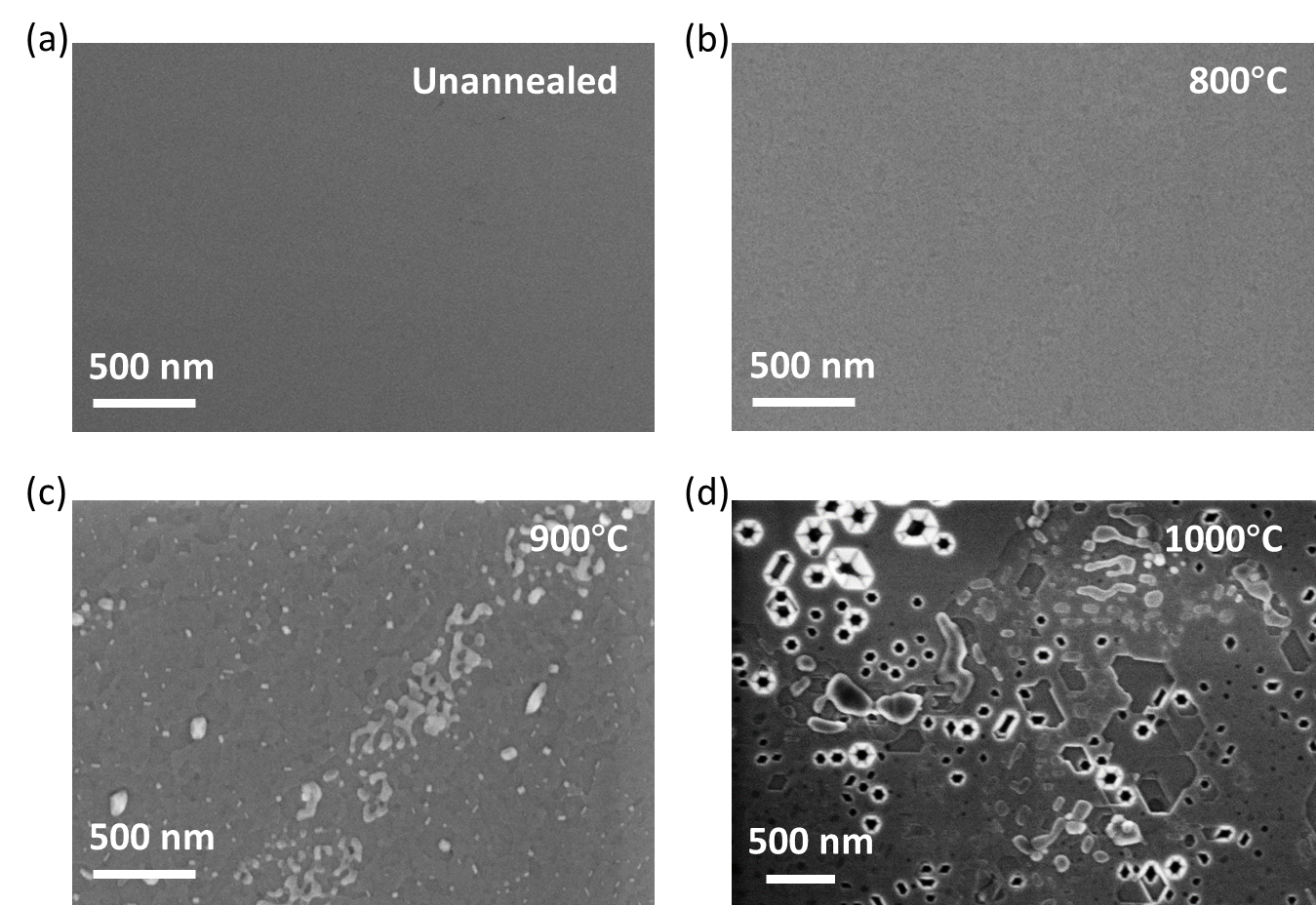


**Figure S7.** SEM images of ZnO areas irradiated with the ion fluence of 6×10^15^ cm^-2^ followed by **(a)** no annealing treatment, and **(b-d)** annealing in air for 40 mins at 800 °C, 900 °C, and 1000 °C, respectively.

**Section 5. Spectroscopic ellipsometry data acquisition and analysis of Ga:ZnO**

We performed infrared ellipsometry measurements (implemented using IR-VASE Mark II ellipsometer, J. A. Woollam) on Ga:ZnO samples for incident angles of 50°, 60°, and 70°. To extract refractive index of the material, a multi-layer model must be built to solve for the inverse problem: find the right $n$ and $\kappa$ and thickness of each layer to fit the measured $\Psi$ and $\Delta$. Note that in our modeling, the $n$ and $\kappa$ are correlated by the Kramers-Kronig relation and usually described by a series of optical oscillator functions such as Lorentz, Sellmeier, Drude, Gaussian, Cauchy, etc.

First, we characterized optical properties of the pristine ZnO substrate using a series of Gaussian oscillators (Table S1 and Fig. S7) expressed as [S9]:

$$\varepsilon_{\mathrm{Gaus}}=\varepsilon_{1}+i\varepsilon_{2}$$

$$\varepsilon_{2}=A_{n}e^{\left( \frac{E-E_{n}}{\sigma} \right)^{2}}-A_{n}{e^{-\left( \frac{E+E_{n}}{\sigma} \right)}}^{2}$$

$$\varepsilon_{1} =\frac{2}{\pi}P\int_{0}^{\infty} \frac{\xi\varepsilon_{2}(\xi)}{\xi^{2}-E^{2}}d\xi$$

Where, $\sigma=\frac{Br_{n}}{2\sqrt{\ln\left( 2 \right)}}$, $Br_{n}=FWHM$, $P$ is the Cauchy Principal Value.

**Table S1.** Fitting parameters of Gaussian oscillators used for pristine ZnO substrate.

| Oscillator | Type | Amplitude $A_{n}$ | Center Energy $E_{n}$ (eV) | Broadening $Br_{n}$  (eV) |
| --- | --- | --- | --- | --- |
| 1 | Gaussian | 85.165 | 0.0506 | 0.00210 |
| 2 | Gaussian | 16.737 | 0.05019 | 0.00610 |
| 3 | Gaussian | 0.32918 | 0.08231 | 0.03047 |
| 4 | Gaussian | 0.05138 | 0.17235 | 0.03051 |
| 5 | Gaussian | 0.21273 | 0.05917 | 0.01021 |
| 6 | Gaussian | 0.11585 | 0.19682 | 0.08180 |
| 7 | Gaussian | 0.03799 | 0.11703 | 0.01615 |

**
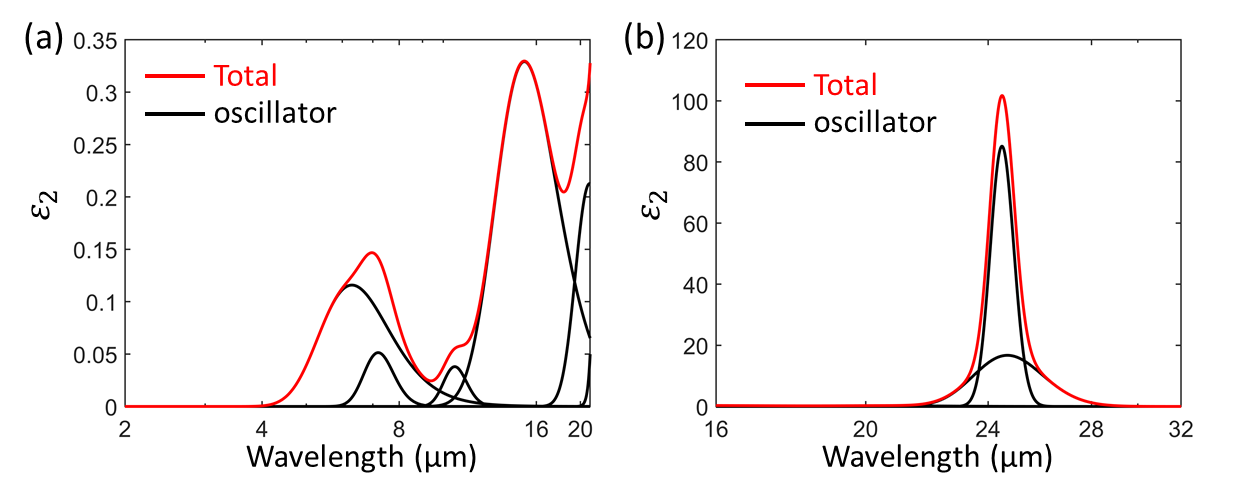
**

**Figure S8.** The fitting $\varepsilon_{2}$ values of the pristine ZnO substrate. The summed $\varepsilon_{2}$ function (red) and individual Gaussian oscillators (black) are plotted in **(b)** from 2 to 20 µm, and **(c)** from 16 to 32 µm.

As shown in Fig. S8 (a, b) the measured (discrete points) and fitted (continuous curves) $\Psi$ and $\Delta$ for the pristine ZnO are in good agreement. Our modeling was also verified by comparing the calculated reflection based on our ellipsometry model to our FTIR reflectance measurements, as shown in Fig. S8(c). Fig. S8(d) plots the extracted complex refractive index of the pristine ZnO substrate.


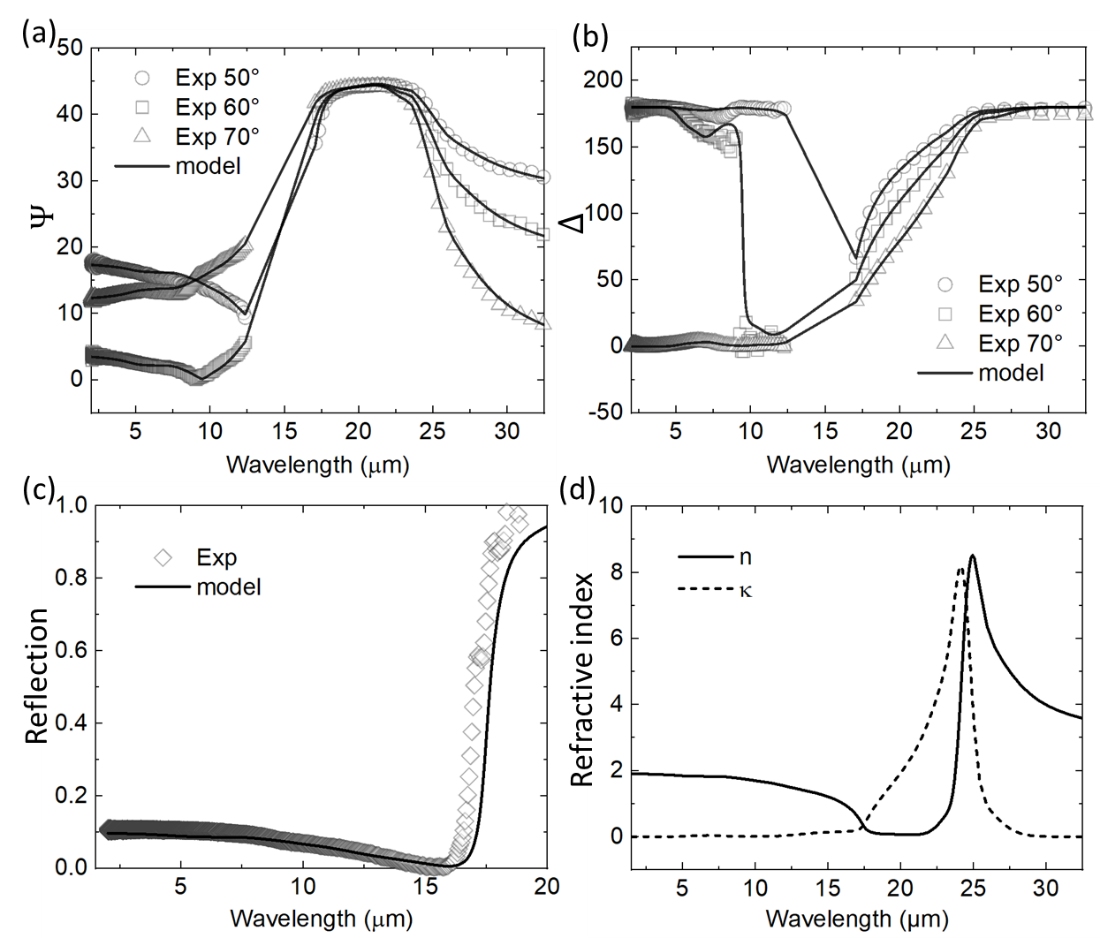


**Figure S9.** **(a, b)** Comparison between fitting (solid curves) and measured ellipsometry data of $\Psi$ and $\Delta$ for the pristine ZnO substrate. **(c)** Comparison between calculated reflectance based on our ellipsometry fitting (solid curve) and FTIR measurements (diamond dots) for the pristine ZnO substrate. **(d)** Extracted complex refractive index of the pristine ZnO substrate.

Then, we followed the same fitting procedures to extract the optical properties of Ga:ZnO samples. For the thermal annealed Ga:ZnO samples, according to the SIMS depth profile shown in Fig. 2(d) in the main text, we found that there is a diffusion plateau underneath the top surface. Therefore, we built two layers to model the top surface and the diffusion layer separately, as discussed in the main text. Each layer includes an additional Drude function to account for the induced carrier concentration due to the FIB-assisted doping (note that the seven Gaussian oscillators are fixed, and only the Drude term was fitted.):

$$\varepsilon_{\mathrm{Drude}}=\varepsilon_{1}+i\varepsilon_{2}=\frac{-\hbar^{2}}{\varepsilon_{0}\rho_{n}(\tau_{n}\cdot E^{2}+i\hbar E)}$$

$$\rho_{n}=\frac{m^{*}}{Nq^{2}\tau}=\frac{1}{q\mu N}$$

Where, $m^{*}$ is the effective mass, $\rho$ is the resistivity, and $\tau$ is the scattering time.

For the 3.1-at.% Ga-doped ZnO sample, we have six fitting parameters, including two sets of thickness, carrier concentration, and mobility: one set for the diffusion layer, and one set for the high carrier concentration layer. Fitting results and comparison between the measured and fitted $\Psi$ and $\Delta$ have been shown in Fig. 2 in the main text.

For the unannealed Ga:ZnO sample, we assumed a thin top layer (on the order of 10 nm, but the thickness was a fitting parameter) described by fixed Gaussian oscillators that describe intrinsic ZnO and an additional Drude term, with a semi-infinite undoped ZnO substrate. Note that this is similar to our fitting procedure with the annealed samples, but with no diffusion layer. The fitting results are shown below. Our fitting yielded a 20-nm layer with carrier concentration of ~4.7$\times$10^20^ cm^-3^ and a mobility of ~11.5 cm^2^/V∙s. Therefore, we observe a substantial carrier activation happening within this thin layer even in the absence of annealing, and the layer’s optical properties can be described by a Drude model.


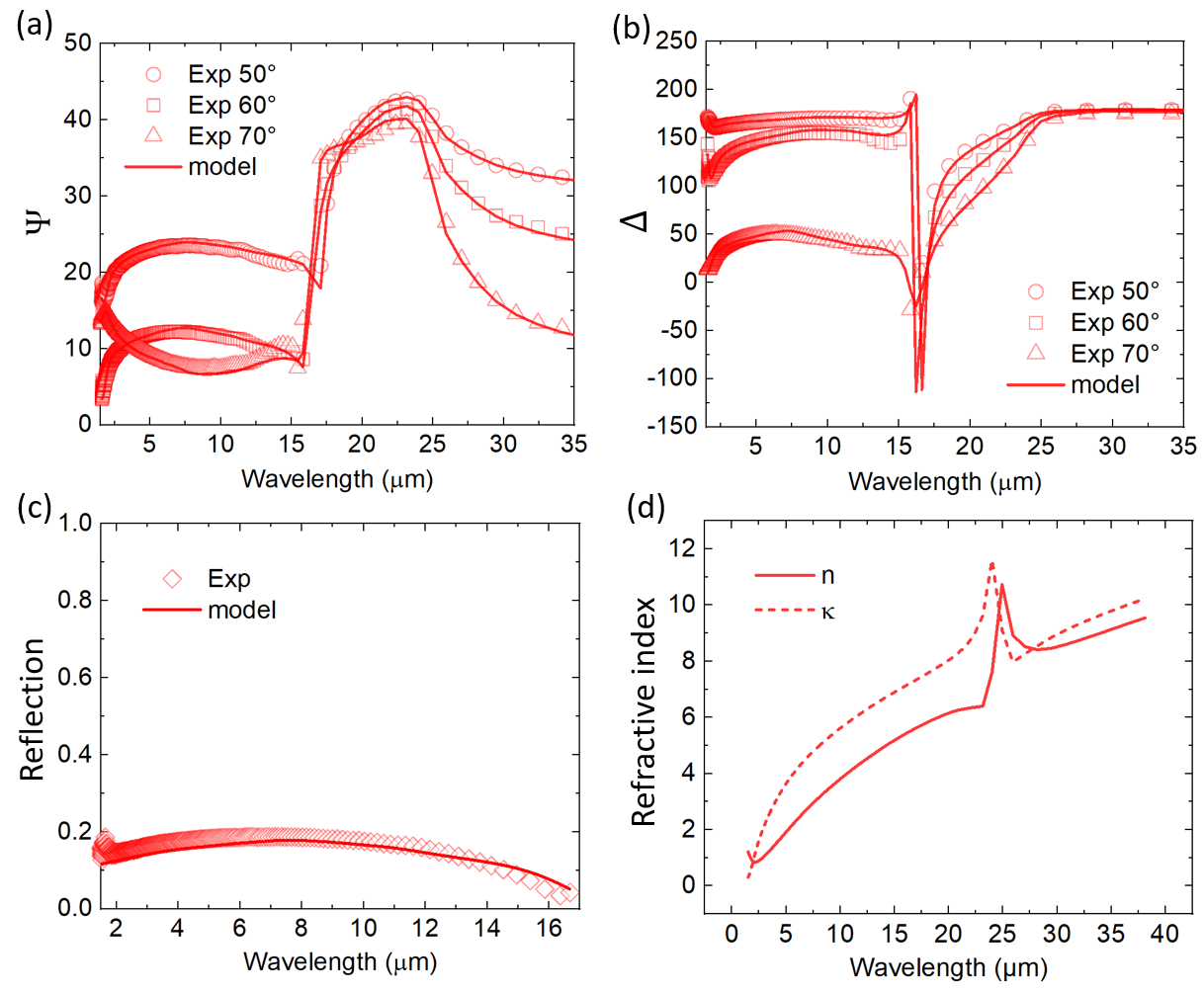


**Figure S10. (a, b)** Comparison between fitting (solid curves) and measured ellipsometry data of $\Psi$ and $\Delta$ for the unannealed Ga:ZnO irradiated by a 30 keV ion accelerator with an ion fluence of 3.6 $\times$ 15 cm^-2^ (corresponding to Ga peak concentrations of 3.1 at.%). **(c)** Comparison between calculated reflectance based on our ellipsometry fitting (solid curve) and FTIR measurements (diamond symbols) for the unannealed Ga:ZnO sample. **(d)** Extracted complex refractive index of the Ga:ZnO layer in the unannealed sample.

In the fitting process, a uniqueness test was performed to check the robustness of the fitting for different parameters. The ellipsometry fitting software WVASE™ uses the mean-squared error (MSE) as the figure of merit, which represents the quality of the match between the data calculated from the model and the experimental data. One way to examine whether the fitting of a parameter is accurate and unique is to take the best-fit model, slightly change the parameter of interest and fix its value (not fitting), and then refit all the other parameters around its value [S9].

Here the parameter of interest is the thickness of the top Ga:ZnO layer. Figure S11 shows the plot of the MSE values versus fixed thickness from 5 nm to 30 nm for unannealed sample, and samples annealed at 800 °C and 900 °C. We can observe that the thickness of 20 nm yields the minimum MSE for the unannealed Ga:ZnO, indicating a good agreement with the TRIM results. As shown in Figure S11(b,c), a thickness of 8 nm yields the minimum MSE for the samples annealed at 800 °C and 900 °C samples. This is a bit smaller than the thickness predicted by TRIM. We hypothesize that the top layer may become thinner due to diffusion of some of the dopants deeper into the sample, and we also suspect that the annealing treatment might push some of the dopants to the surface. However, it is also possible that the fitted thickness is imperfect because in reality there is a gradient of dopants through the material, rather than two homogeneous layers as assumed in the fit.


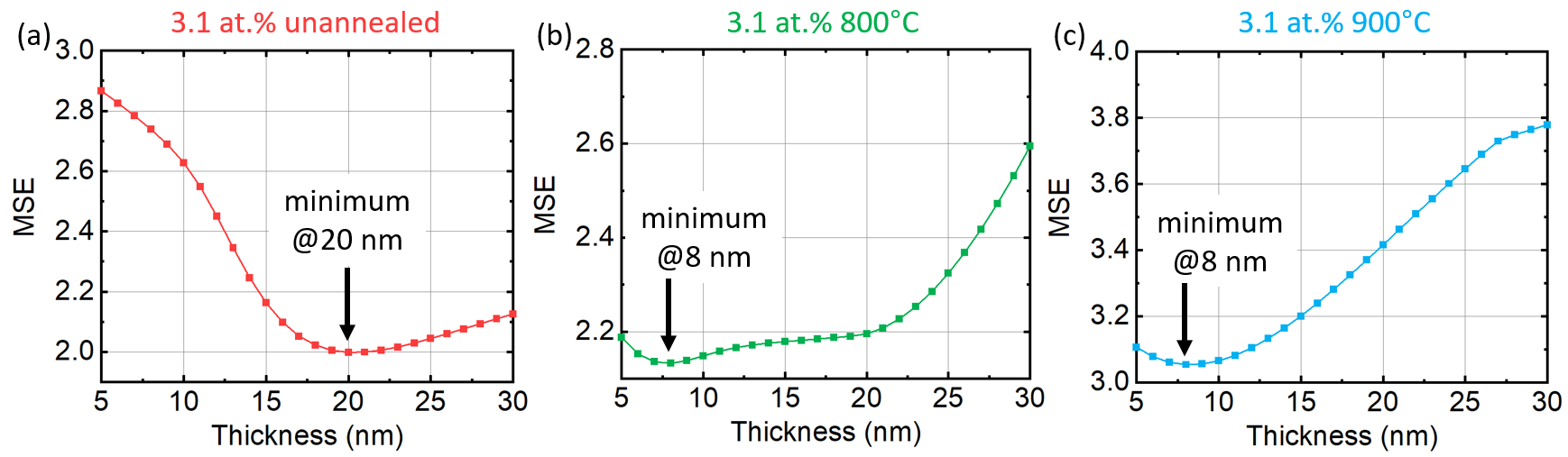


**Figure S11. (a-c)** Ellipsometry fitting uniqueness test for the thickness of the top layer of the unannealed Ga:ZnO, and for Ga:ZnO annealed at 800 °C and 900 °C, respectively.

**Section 6. Implantation parameters of FIB-VO_2_ and SEM images**

We took SEM images of the FIB-VO_2_ sample. As shown in Fig. S12, FIB-irradiated regions have a contrast compared to pristine regions, but there is no obvious change of the morphology caused by the FIB irradiation in comparison with the pristine VO_2_ region, as seen in the high-resolution SEM Figure S12(e).


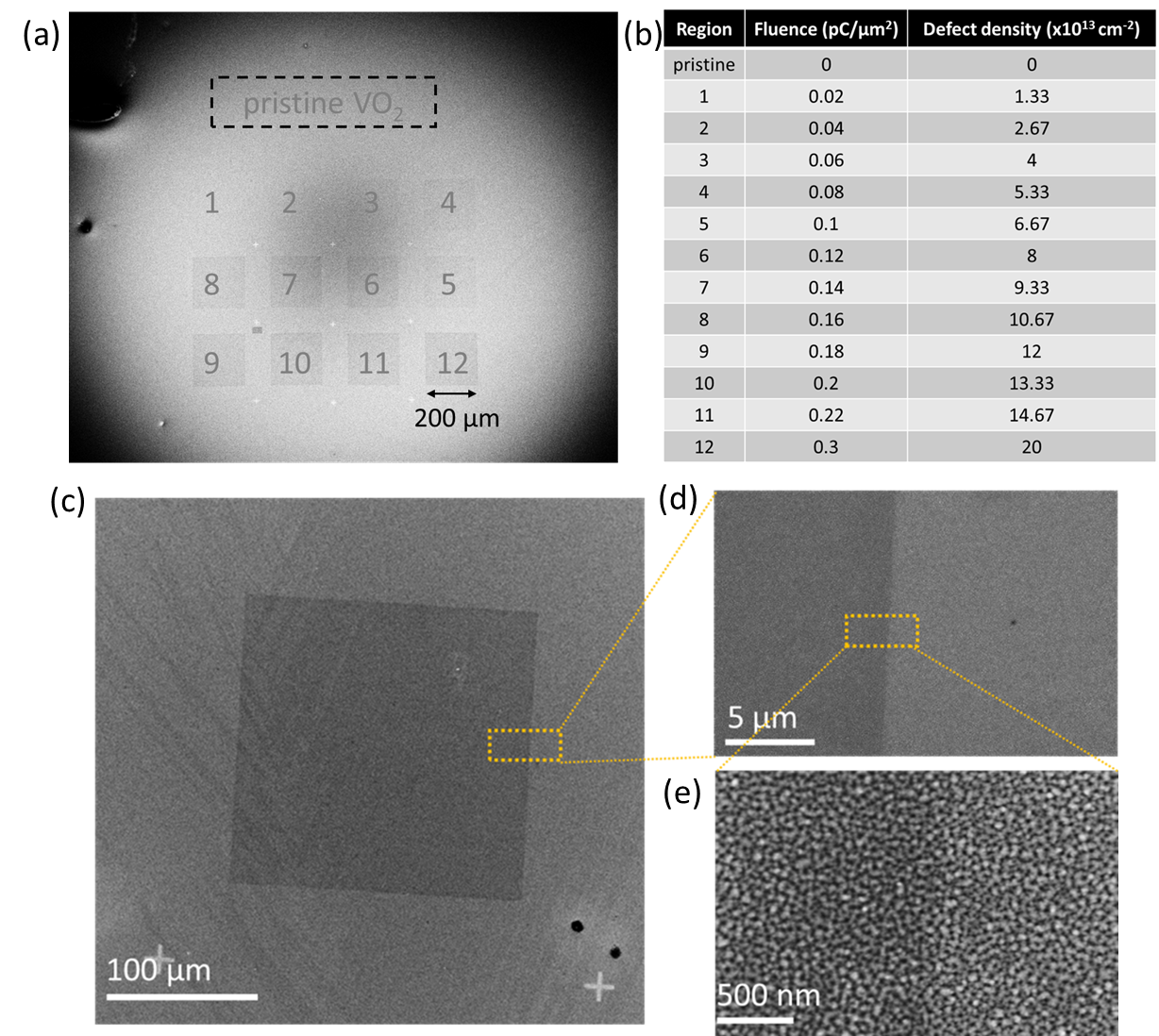


**Figure S12.** **(a,b)** SEM image of the top surface of the FIB-VO_2_ sample with the corresponding ion fluences given in the table. **(c-e)** SEM images of the FIB-irradiated VO_2_ region #12 with an ion fluence of 2 $\times$14 cm^-2^.

We performed Raman mapping to show that FIB is able to locally modulate the IMT of VO_2_ within sub-micrometer areas. We used an excitation laser of 532 nm with a step size of 1 μm of a 5-by-40-μm rectangular region that included both pristine and irradiated regions. As shown in Fig. S13, the irradiated VO_2_ region was transformed to the metallic phase at 50 °C, featuring no Raman mode at 610 cm^-1^, while the pristine region was still in its insulating phase, with a strong Raman mode at 610 cm^-1^.


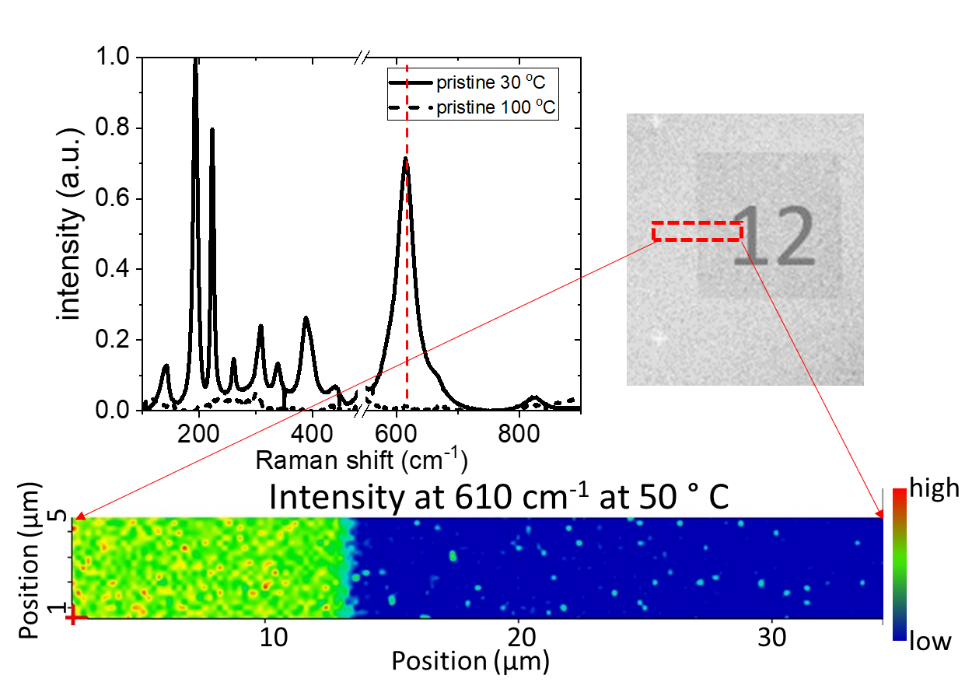


**Figure S13.** Raman mapping across the boundary between a pristine region and a region irradiated with fluence of 2 $\times$ 14 cm^-2^.

We also took SEM images of cross section of the FIB-VO_2_ sample. The cross section was created by a FIB-assisted milling process (implemented using FIB-SEM, Zeiss Auriga), as shown in Fig S14. In order to obtain clear imaging of the VO_2_ boundaries, we pre-deposited ~300-nm thick, ~1-by-1-mm patch of copper (Cu) on top of the VO_2_ and milled through the layers within this Cu patch area, resulting in a clean cross section of the VO_2_ layer for thickness measurement. The thickness of VO_2_ was measured to be $(52 \pm5)$ nm, after we compensated the angle of 54° between the FIB and SEM beam.

**
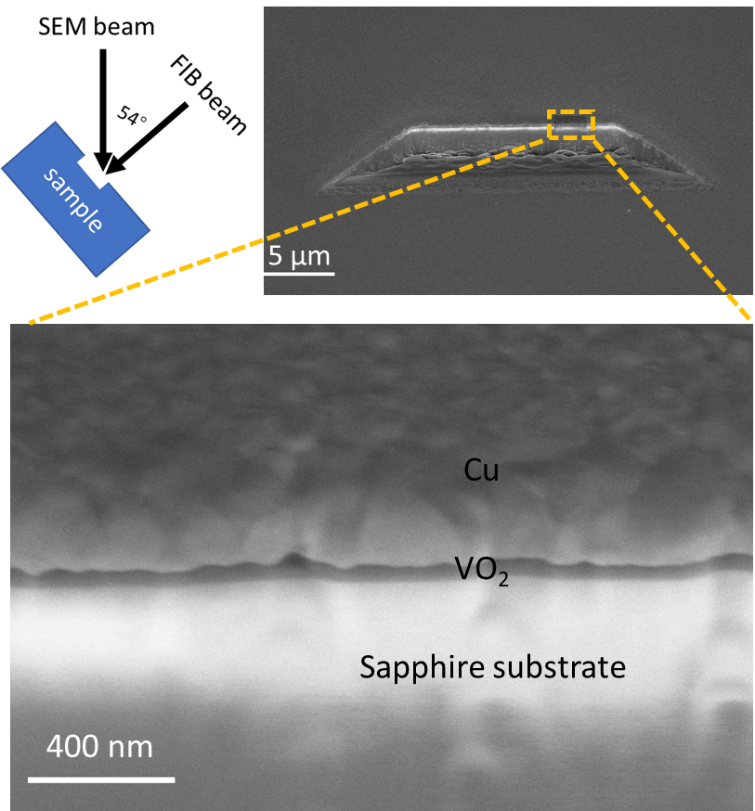
**

**Figure S14.** Schematic of FIB-milling-assisted cross-section imaging of the FIB-VO_2_ sample. The measured thickness of VO_2_ is (52$\pm$5) nm.

**Section 7. Full dataset of optical refractive indices of FIB-VO_2_ for different ion fluences**

In Fig. 4(c) of the main text, we only plotted the characterized refractive indices of the FIB-VO_2_ for a single wavelength of 9 µm to clearly show the evolution of refractive-index values versus temperature and ion fluence. Fig. S15 includes refractive indices of VO_2_ FIB-irradiated by different fluences, for wavelengths of 6 – 14 µm.

**
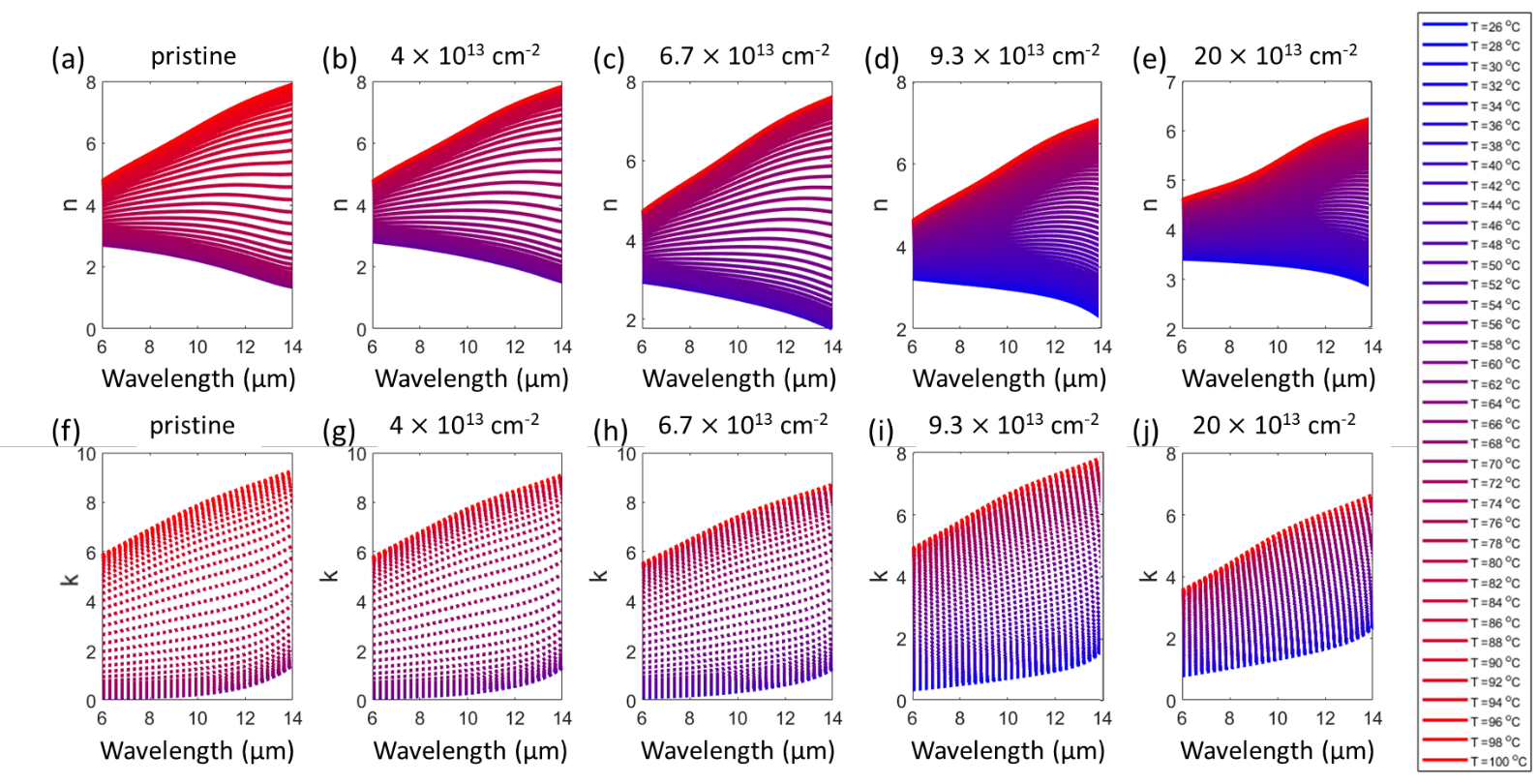
**

**Figure S15.** Full dataset of **(a-e)** real and **(f-j)** imaginary parts of temperature-dependent refractive indices of VO_2_ irradiated with different ion fluences as indicated on top of each figure, for wavelengths from 6 to 14 µm.

**Section 8. Stability of the hysteresis curves after multiple thermal cycles**

To check the stability of the hysteresis curves after multiple thermal cycles, we ran temperature-dependent FTIR reflectance measurements on region #6 (irradiated with the ion fluence of 8$\times$13 cm^-2^) of our FIB-VO_2_ sample for 3 cycles of heating and cooling, as shown in the figure below. Our results show that the hysteresis and IMT temperature is stable with multiple thermal cycles between room temperature and 90 °C.

**Figure S16.** FTIR reflectance measurements on FIB-VO_2_ for 3 cycles of heating and cooling between room temperature and 90 °C.

**References**

[S1] J. F. Ziegler and J. P. Biersack, “The stopping and range of ions in matter,” in *Treatise on heavy-ion science*, Springer, 1985, pp. 93–129.

[S2] F. Mitsugi, Y. Umeda, N. Sakai, and T. Ikegami, “Uniformity of gallium doped zinc oxide thin film prepared by pulsed laser deposition,” *Thin Solid Films*, vol. 518, no. 22, pp. 6334–6338, Sep. 2010, doi: 10.1016/j.tsf.2010.03.044.

[S3] J. Moulder, W. Stickle, W. Sobol, and K. D. Bomben, “Handbook of X-Ray Photoelectron Spectroscopy,” *undefined*, 1992, Accessed: Dec. 28, 2021. [Online]. Available: https://www.semanticscholar.org/paper/Handbook-of-X-Ray-Photoelectron-Spectroscopy-Moulder-Stickle/6165d59e158c88267b1154c167da68bfca644f4a

[S4] A. Shchukarev and D. Korolkov, “XPS Study of group IA carbonates,” *Open Chemistry*, vol. 2, no. 2, pp. 347–362, 2004, doi: 10.2478/BF02475578.

[S5] M. Chen *et al.*, “X-ray photoelectron spectroscopy and auger electron spectroscopy studies of Al-doped ZnO films,” *Applied Surface Science*, vol. 158, pp. 134–140, May 2000, doi: 10.1016/S0169-4332(99)00601-7.

[S6] C.-Y. Tsay, K.-S. Fan, and C.-M. Lei, “Synthesis and characterization of sol–gel derived gallium-doped zinc oxide thin films,” *Journal of Alloys and Compounds*, vol. 512, no. 1, pp. 216–222, Jan. 2012, doi: 10.1016/j.jallcom.2011.09.066.

[S7] A. E. Rakhshani, A. Bumajdad, J. Kokaj, and S. Thomas, “Structure, composition and optical properties of ZnO:Ga films electrodeposited on flexible substrates,” *Appl. Phys. A*, vol. 97, no. 4, p. 759, Aug. 2009, doi: 10.1007/s00339-009-5362-4.

[S8] V. R. Shinde, T. P. Gujar, C. D. Lokhande, R. S. Mane, and S.-H. Han, “Mn doped and undoped ZnO films: A comparative structural, optical and electrical properties study,” *Materials Chemistry and Physics*, vol. 96, no. 2, pp. 326–330, Apr. 2006, doi: 10.1016/j.matchemphys.2005.07.045.

[S9] J. Woollam, “Guide to Using WVASE 32: Spectroscopic Ellipsometry Data Acquisition and Analysis Software,” *JA Woollam Company, Lincoln, NE, USA*, 2012.
